# Evaluation of Nutraceutical Properties of Eleven Microalgal Strains Isolated from Different Freshwater Aquatic Environments: Perspectives for Their Application as Nutraceuticals

**DOI:** 10.3390/foods11050654

**Published:** 2022-02-23

**Authors:** Carolina Chiellini, Valentina Serra, Leandro Gammuto, Adriana Ciurli, Vincenzo Longo, Morena Gabriele

**Affiliations:** 1 Institute of Agricultural Biology and Biotechnology (IBBA), Italian National Research Council, Via Moruzzi, 1, 56124 Pisa, Italy; carolina.chiellini@ibba.cnr.it (C.C.); vincenzo.longo@ibba.cnr.it (V.L.); 2 Department of Biology, University of Pisa, Via A. Volta 4/6, 56126 Pisa, Italy; valentinasrr@gmail.com (V.S.); leandro.g@hotmail.it (L.G.); 3 Department of Agriculture, Food and Environment (DAFE), University of Pisa, Via del Borghetto 80, 56124 Pisa, Italy; adriana.ciurli@unipi.it

**Keywords:** microalgae, nutraceuticals, novel foods, antioxidants, antioxidant activity, bioactive compounds

## Abstract

The increasing global population and the simultaneous growing attention to natural, sustainable, and healthier products are driving the food industry towards research on alternative food sources. In this scenario, microalgae are gaining worldwide attention as “functional feedstocks” for foods, feeds, supplements, and nutraceutical formulations, being a source of high-value metabolites including polyphenols and other antioxidant compounds. In this work, eleven microalgal strains from freshwater environments were evaluated for their nutraceutical properties, focusing on photosynthetic pigments, total polyphenols, and flavonoid content, as well as *in vitro* antioxidant activities. Data helped to select those strains showing the most promising features for simultaneous massive growth and bioactive compound production. Results highlighted that the microalgae have variable values for both biochemical parameters and antioxidant activities, mainly depending on the solvents and applied treatment rather than on the isolation sources or the phylogenetic attribution. According to our results, the putative best candidates for massive cultivation under laboratory conditions for the simultaneous extraction of different molecules with nutraceutical potential are strains F1 (Scenedesmaceae), F3 (*Chlamydomonas debariana*), R1 (*Chlorella sorokiniana*), and C2 (*Chlorella*-like).

## 1. Introduction

The increasing global population directly affects the demand for food and energy. Currently, it is estimated that, by 2030, the population will rise to approximately 9 billion people, and 12% of those people will be facing the challenge of food scarcity [1]. For this reason, food crops are probably not sufficient to satisfy the food demand of the human population [2]. In this scenario, there is an urgent need for research on new sources of sustainable foods, which might be represented by microalgae.

Microalgae are unicellular photosynthetic microorganisms, almost ubiquitous in all environments, especially in freshwater and seawater, but also in soil and air. Microalgae can grow in different climatic conditions, and, analogously to plants, they can use sunlight energy to fix atmospheric CO_2_, converting it into biomass through photosynthesis [3]. While growing, the microalgal population accumulates lipids, carbohydrates, proteins, essential amino acids, and high-value metabolites, including pigments, polyunsaturated fatty acids (PUFAs), vitamins, and other natural anti-inflammatory and antioxidant compounds [4,5]. Consequently, the microalgal biomass is a potential feedstock for the production of high-quality food/feed and nutraceuticals. Among the advantages in the use of microalgae as a feedstock, there are the fast growth rates, the ability to adapt to a wide range of climatic conditions, and the high CO_2_ uptake efficiency [6]. These characteristics are specific to the different microalgal species [7].

Despite the great microalgal biodiversity over the Earth, only a small percentage of these microorganisms are currently studied and characterized for commercial and research purposes [2]. Historically, microalgae for food purposes were used among ancient populations [4,8]; from AD 1300, Aztecs consumed Spirulina (*Arthrospira platensis*, *Arthrospira maxima*). In addition, the populations in Chad used daily Spirulina for food purposes as well, while *Nostoc* sp. species were commonly consumed in China, Mongolia, Tartaria, and South America. Finally, the filamentous green algae *Spirogyra* and *Oedogonium* are also used as a dietary component in Burma, Thailand, Vietnam, and India [8]. Only in 1974, Spirulina was declared the best food for the future by the United Nations World Food Conference [8]. Currently, *Chlorella* and Spirulina are widely commercialized and used both for human nutrition and to feed many types of animals. Additionally, other microalgal species (e.g., *Tetraselmis*, *Isochrysis*, *Pavlova*, *Phaeodactylum*, *Chaetoceros*, *Nannochloropsis*, *Skeletonema*, and *Thalassiosira* spp.) are also used as feeds in aquaculture [9].

Several studies have pointed out the beneficial role of a diet enriched in antioxidants in preventing the onset and slowing the progression of several diseases [10,11,12]. Oxidative stress underlies certain chronic and degenerative diseases, such as cancer and cardiovascular and neurodegenerative disorders [13,14]. For these purposes, in the last several decades, many studies have focused on the use of microalgae in nutraceutical applications [8,15], especially considering them as a whole-food ingredient. Another alternative application of such microorganisms in the field of nutrition is the extraction of food ingredients and nutraceutical compounds from microalgal cultures to allow the more targeted use of their nutritional and functional properties [16]. Other than being cost-effective, the extraction process must be capable of recovering high yields of the targeted components and, at the same time, avoiding the degradation and contamination of the final products [16]. Currently, the most challenging part within the process of microalgal use for nutraceutical purposes deals with the separation, recovery, and treatment of the biomass; accordingly, among the most promising technologies for the extraction and concentration of the biologically active compounds from the culture media, there are those based on the use of membranes, although the improvement of techno-economical aspects is still being investigated [17].

Among secondary metabolites, chlorophyll is a photosynthetic pigment found in plants, algae, and cyanobacteria, and its production by microalgae is variable depending on the culture conditions [18]. In the field of nutraceuticals, chlorophyll can be applied as a colorant of natural origin for food [19], and its beneficial role as an antitumor agent has been described as well [20]. Carotenoids, instead, are terpenoid pigments derived from tetraterpenes that are synthesized by plants, algae, bacteria, and some species of fungi [21], and play a key role during photosynthesis by scavenging ROS, especially singlet oxygen [22]. Indeed, microalgae can accumulate secondary carotenoids in lipid bodies under stressful conditions, such as light excess, increased salinity, low or high temperature, UV-B irradiation, and nutritional stress (e.g., nitrogen deficiency). Among others, polyphenols represent secondary metabolites naturally occurring in several vegetables, fruits, cereals, flowers, microalgae, etc., with convenient health benefits [10,23,24]. These compounds confer many features to foods, including color, odor, flavor, and bitterness, and are classified into phenolic acids, flavonoids, lignans, and stilbenes, according to the number and arrangement of phenol rings and functional groups [24]. Moreover, polyphenols, mostly flavonoids, are well-characterized health-promoting compounds as they prevent the onset and progression of many oxidative stress-based diseases by radical scavenging activity, being donors of electrons or hydrogen atoms, and by inhibiting iron-mediated oxyradical formation [14,25]. Furthermore, these compounds exhibit several pharmacological properties, such as anti-inflammatory, antitumor, antimicrobial, and antiviral activities [10,25].

Nowadays, microalgae are gaining worldwide attention as sources of high-value metabolites, antioxidants, and anti-inflammatory molecules and represent promising feedstocks in food, feed, supplement, and health product preparation [5,23,26]. Several studies have evidenced the nutraceutical potential of microalgae in fighting obesity, improving immunity and neurological development, lowering cholesterol and blood pressure, and maintaining good heart condition [5,27,28]. Moreover, other studies have noted their anticancer, antibacterial, and antiviral properties and positive effects on diabetes, anemia, gastric ulcers, and constipation [27,29,30,31]. Therefore, microalgae could represent “functional ingredients” in food preparation as they provide beneficial effects on human health and wellness [5,27,28,29,30,31]. Moreover, microalgae might represent natural alternatives to manage and prevent several diseases, with limited side effects.

In this work, eleven autochthonous and newly isolated microalgal strains from freshwater environments were evaluated for their nutraceutical properties, focusing on the photosynthetic pigments, total polyphenols, and flavonoid content. Moreover, the *in vitro* antioxidant activity of each microalgal strain was screened by FRAP (ferric reducing antioxidant power) and DPPH (2,2-diphenyl-1-picrylhydrazyl) assays.

Our data revealed the presence of variable content of both biochemical parameters and antioxidant activities, depending mainly on the solvent type and applied treatment rather than on the isolation sources or the phylogenetic attribution of the microalgal strains. Among these strains, this work aimed to select those showing the most promising features for simultaneous massive growth and bioactive compound production.

## 2. Materials and Methods

### 2.1. Microalgal Strain Isolation

#### 2.1.1. Sample Collection

Eleven different microalgal strains were characterized and evaluated for their nutraceutical properties. Four of these strains were taxonomically related to *Chlorella sorokiniana* [32], and were collected from Lake Massaciuccoli (namely strains Idr, CL_Sc, CL_Ch, and FB). The strain SEC_Li_ChL_1 was isolated from an artificial lake in Rosignano Marittimo (LI) and was taxonomically identified as part of the *Chlorella–Micractinium* clade [33]. These five strains were kindly provided from the collection of the Dept. of Agriculture Food and Environment of the University of Pisa. The remaining six strains are currently part of the collection of the laboratory of the Institute of Agricultural Biology and Biotechnology, Italian National Research Council, located in Pisa, and are characterized in this work for the first time. Strains F1, F2, F3, and F4 were isolated from a water sample collected in the Fucecchio Marshland, which is the largest inland Italian swamp. This area comprises approximately 2000 hectares, and it is situated between the provinces of Florence, Prato, Pistoia, Lucca, and Pisa. More precisely, the water sample from which the four strains were isolated was collected close to the protected area of “Le Morette” (43°48′31″ N 10°48′18″ E). The strain R1 was isolated from a water sample collected in the pot of a plant placed on a private terrace in Pisa. The last microalgal strain, C2, was isolated from the heating system of a private house in Empoli (Florence). For strains F1, F2, F3, F4, and R1, water samples were collected in 50 mL sterile falcon tubes, and immediately brought to the laboratory for processing. The strain C2 was collected from a transparent plastic container located on the outside of a boiler that was external to a house on a terrace; the sample contained polyphosphate salts, which serve as a softener for the water that passes through the whole system. A greenish biofilm was observed in the plastic container, on the side exposed to the polyphosphate solution. This biofilm was collected with a sterile swab in September 2020; the swab was immediately brought to the laboratory for microalgal isolation procedures.

#### 2.1.2. Isolation of Microalgal Strains from Collected Samples

Each water sample collected for F1, F2, F3, F4, and R1, together with the C2 biofilm previously dissolved in 100 mL Tris–Ammonium Phosphate (TAP) medium, was subjected to a procedure of enrichment by dilution with TAP medium [34] (1/5 *v*/*v*). The TAP medium was prepared as follows: Tris base 2.00 10^−2^ M, 25 mL L^−1^ Beijerinck salts (15 g L^−1^ NH_4_Cl, 4 g L^−1^ MgSO_4_ 7H_2_O, and 2 g L^−1^ CaCl_2_ 2H_2_O), 1 mL L^−1^ phosphate solution (28.8 g 100 mL^−1^ K_2_HPO_4_, and 14.4 g 100 mL^−1^ KH_2_PO_4_), 1 mL L^−1^ acetic acid and 1 mL L^−1^ Hutner trace elements (5.00 g 100 mL^−1^ Na_2_EDTA 2H_2_O (Titriplex III), 2.20 g 100 mL^−1^ ZnSO_4_ 7H_2_O, 1.14 g 100 mL^−1^ H_3_BO_3_, 0.50 g 100 mL^−1^ MnCl_2_·4H_2_O, 0.50 g 100 mL^−1^ FeSO_4_ 7H_2_O, 0.16 g 100 mL^−1^ CoCl_2_ 6H_2_O, 0.16 g 100 mL^−1^ CuSO_4_ 5H_2_O, and 0.11 g 100 mL^−1^ (NH_4_)6MoO_3_). All reagents were purchased from Fluka-Sigma-Aldrich, Inc. (St. Louis, MO, USA).

The enriched samples were maintained for two weeks in a growth chamber under a controlled temperature (24/22 °C), and under a 16/08 h day–night cycle with a Photosynthetic Photon Flux Density (PPFD) of 70 μmol photons m^−1^ s^−1^. After two weeks, some replicates (100 μL each) of the enriched water samples were streaked on TAP agar plates, which were then kept in the growth chamber as described above. This process was further repeated until a single morphology indicating the presence of a single strain was visible. Colonies were randomly chosen from the streaked plates and pre-inoculated in a liquid TAP medium (50 mL). Four dense pre-cultures (200 mL) were obtained from the samples collected in Fucecchio, and one dense pre-culture was recovered from the sample collected on the private terrace, by adding fresh sterile TAP medium every week to the pre-inocula. Strains F1, F2, F3, F4, C2, and R1 were taxonomically identified, as described in Section 2.2.

#### 2.1.3. Cultivation of Microalgae

All the microalgal cultures were maintained in TAP medium under laboratory conditions (controlled temperature of 24/22 °C, and a 16/08 h day–night cycle with PPFD of 70 μmol photons m^−1^ s^−1^). The exhausted medium was replaced with a sterile fresh one roughly every two weeks. The cultures were periodically observed under a light microscope (Carl Zeiss Axioskop 20 EL-Einsatz 451487), and the general health status of the culture was monitored to ensure the absence of any eukaryotic contamination (monoclonal cultures). The maintenance of the microalgal cultures (e.g., medium replacement, microscopic observations, etc.) was performed in sterile conditions, under biological flow, and using sterilized plastic, glass, and medium. Despite the sterility that occurred during the maintenance procedure, we cannot a priori exclude the association of the microalgal strains with symbiotic phycospheric bacteria and, hence, the axenicity of the microalgal cultures.

### 2.2. Molecular Identification of the Microalgal Strains

Six of the eleven microalgal strains used in this work were not previously characterized or published (F1, F2, F3, F4, C2, and R1); accordingly, a molecular characterization based on the sequencing of the 18S and/or the ITS rRNA sequences was performed, as described in Ciurli et al. [33]. Briefly, total genomic DNA was extracted following the protocol described by Saba et al. [35], starting from a fresh pellet obtained by centrifugation of 2 mL of each liquid monostrain microalgal culture (5′ at 14,000× *g*—Eppendorf 5415R). The 18S rRNA and the fragment comprising the final portion of 18S, the complete ITS1-5.8S-ITS2, and the initial portion of 28S were amplified as described in Ciurli et al. [33], using a C1000™ Thermal Cycler (Bio-Rad, CA, USA). Amplicons obtained from F2 and F3 and C2 microalgal cultures were purified by ethanol/EDTA/Na–acetate precipitation, according to Ciurli et al. [33], and they were sequenced by the company “BMR Genomics” (Padova, Italy). Amplicons obtained from F1, F4, and R1 strains were purified with the “Cycle Pure Kit” from Euroclone (Italy) and they were sequenced by the company Eurofins Genomics (Germany). All the obtained sequences were deposited in GeneBank under the accession numbers listed in Table 1. The obtained DNA sequences were separately subjected to NCBI Blast analysis [36] to determine their preliminary affiliation through comparison with all the sequences present in the international databases.

### 2.3. Microalgal Sample Extraction

Four different types of solvent extraction (acetone, methanol, ethanol, and distilled water) were carried out and all the analyses were evaluated, for each strain, on both fresh and dried biomass. For the photosynthetic pigments, only the extractions with methanol, ethanol, and acetone were considered, being extracted with organic solvents and not with water [37]. The dried biomass was obtained by centrifuging 50 mL of microalgal culture (3000× *g* 10 min, Jouan CR 3i centrifuge), removing the supernatant, and drying the pellet at 40 °C in a thermoblock for 15–20 h. For each solvent, the dried biomass of each strain was weighed in a Kern ACJ/ACS 220-AM analytical balance and resuspended to a final concentration of 5 mg mL^−1^ dried biomass. The fresh biomass was obtained by centrifugation of 15 mL fresh microalgal culture (3000× *g* 10 min, Jouan CR 3i centrifuge), and removing the supernatant. Following this, the fresh biomass pellets were resuspended in 10 mL of each solvent. The extractions were performed under dark and shaking conditions for 24 h at room temperature (23 ± 2 °C). For each sample, three replicates were considered. For each strain, 15 mL fresh microalgal culture was dried and weighed (Table S1).

### 2.4. Microalgal Phytochemical Composition

#### 2.4.1. Photosynthetic Pigment Content and Analysis

The total chlorophyll and carotenoids were spectrophotometrically estimated as described in Lichtenthaler [37], using a Perkin Elmer UV/VIS Lambda 365 spectrophotometer (Perkin Elmer Italia, Milano, Italy). The absorbance of microalgal extracts was analyzed with regard to the blank at 665.2, 652.4, and 470.0 nm, and the total chlorophyll and carotenoid content were expressed as µg mL^−1^ according to the equations indicated by Lichtenthaler [37], considering the initial volume of fresh culture, 50 mL and 15 mL, respectively, used for the dry and fresh biomasses.

#### 2.4.2. Total Polyphenol and Flavonoid Content

The microalgal strains were screened for total polyphenol and flavonoid content as previously described [38]. Total polyphenols, estimated as Folin–Ciocalteu (FC) reducing capacity, were expressed as mg of gallic acid equivalents (GAE) g^−1^ dry weight (dw). Briefly, 0.1 mL of microalgal extract was mixed with 0.5 mL of 0.2 N FC reagent and incubated in the dark for 5 min. Then, 0.4 mL of 0.7 M Na_2_CO_3_ was added. After 2 h of incubation at room temperature in the dark, the absorbance was recorded at 760 nm using a Perkin-Elmer Lambda 365 spectrophotometer (Perkin Elmer Italia, Milano, Italy). Total flavonoids were quantified using the aluminum chloride colorimetric method and expressed as mg quercetin equivalents (QE) g^−1^ dw. Briefly, 0.2 mL of microalgal extract was mixed with 0.8 mL of distilled water and 0.06 mL of 5% NaNO_2_ (Carlo Erba, Milan, Italy) and incubated for 5 min at room temperature. Finally, 0.06 mL of 10% AlCl_3_ (Carlo Erba, Milan, Italy) was added and, following 6 min incubation, reactions were neutralized with 0.4 mL of 1 M NaOH and 0.48 mL of distilled water. Absorbance was measured after 30 min at 430 nm using a Perkin-Elmer Lambda 365 spectrophotometer (Perkin Elmer Italia, Milano, Italy). Regarding fresh microalgal cultures, total polyphenol and flavonoid content was calculated based on the dry biomass weight (yield) listed in Table S1. All reagents were purchased from Fluka-Sigma-Aldrich, Inc. (St. Louis, MO, USA), unless otherwise specified.

### 2.5. In Vitro Antioxidant Activities

#### 2.5.1. Ferric Reducing Antioxidant Power (FRAP) Assay

The antioxidant capacity of microalgal strains to reduce ferric iron (Fe^3+^) to ferrous iron (Fe^2+^) was determined by the FRAP assay, as previously reported [39]. Briefly, a solution containing 300 mM acetate buffer (pH 3.6), 10 mM 2,4,6-Tri(2-pyridyl)-s-triazine (TPTZ) in 40 mM HCl, and 20 mM FeCl_3_·6H_2_O (VWR, Milan, Italy) was added to each microalgal extract (85 μL). Following 30 min incubation at room temperature, the absorbance was measured at 593 nm using a Perkin-Elmer Lambda 365 spectrophotometer (Perkin Elmer Italia, Milano, Italy), and results were expressed as Fe^2+^ equivalents (μM) using a water solution of FeSO_4_·7H_2_O (100–2000 μM; VWR, Milan, Italy) used as a standard. All reagents were purchased from Fluka-Sigma-Aldrich, Inc. (St. Louis, MO, USA), unless otherwise specified.

#### 2.5.2. Radical Scavenging Activity

The radical scavenging activity of the microalgal extract was determined using the 2,2-diphenyl-1-picrylhydrazyl (DPPH) assay, as described by Boudjou et al. [40], with some modifications. Briefly, 50 μL of each microalgal extract was added to 1950 μL of methanolic DPPH solution (60 μM) and mixed for 30 min in the dark at 30 °C. The absorbance was recorded at 517 nm using a Perkin-Elmer Lambda 365 spectrophotometer (Perkin Elmer Italia, Milano, Italy). The antiradical activity (ARA) of both fresh and dried microalgal extract, referring to 10 mL culture and 5 mg/mL dried biomass, respectively, was expressed as the percentage of DPPH inhibition using the following equation:

ARA = [1 − (AS/AC)] × 100

where AS is the absorbance of the sample and AC is the absorbance of the control. All reagents were purchased from Fluka-Sigma-Aldrich, Inc. (St. Louis, MO, USA), unless otherwise specified.

### 2.6. Statistical Analysis

The R software [41] was used for the construction of the heatmaps for total chlorophyll, carotenoids, polyphenols, flavonoids, FRAP, and DPPH using the pheatmap package [42]. PCA statistics were performed separately on the dataset related to the dried biomass results and on the fresh culture results; the two data matrices were normalized with the Z-score calculation method, and then analyzed using the PAST software [43]. Pearson correlation analysis on carotenoids, polyphenols, flavonoids, FRAP, and DPPH data was performed using GraphPad Prism, version 5.00 for Windows (GraphPad Software, San Diego, CA, USA); *p*-values < 0.05 were considered statistically significant.

## 3. Results

### 3.1. Molecular Identification of the Microalgal Strains

The results of the molecular characterization of the six newly isolated microalgal strains are reported in Table 1. Considering all eleven microalgae used in this work, six strains were taxonomically related to *Chlorella sorokiniana* (F4 and R1 from Fucecchio Marshland, and FB, CL _Sc, CL _Ch, and Idr from Lake Massaciuccoli); two strains from Fucecchio Marshland were attributed to *Chlamydomonas debaryana* species (F2 and F3), SEC_Li_ChL_1 and C2 were two *Chlorella*-like strains from Rosignano Marittimo and from a private house in Empoli, respectively, while strain F1 from the Fucecchio Marshland belonged to the Scenedesmaceae family.

### 3.2. Quantification of Photosynthetic Pigments

**Figure 1:**
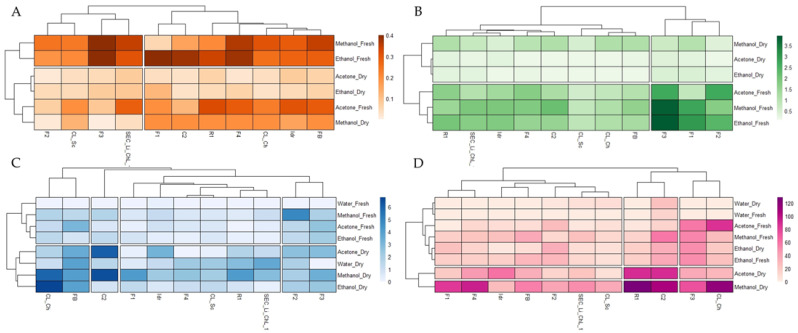
Heatmaps representing the total carotenoid (**A**), total chlorophyll (**B**), polyphenol (**C**), and flavonoid (**D**) content of the microalgal strains under different treatments and solvents. Each datum was obtained from the averaged values of three replicate measures.

The quantification of photosynthetic pigments is reported in Supplementary Tables S2 (total chlorophyll) and S3 (carotenoids). The two treatments (i.e., dried biomass and fresh biomass) determine a difference resulting in all cases in higher pigment content in the fresh biomass for all three solvents used, with the only exceptions represented by the carotenoid content of strains F1, F4, and C2 under methanol treatment, which was higher in the dried biomass than in the fresh one. This evidence is also visible in the two heatmaps (Figure 1A,B), where the clusterization of the strains and of the applied treatment is evident too. Concerning the chlorophyll content (Figure 1B), the main clusterization divided dried and fresh treatments, confirming the greater content of chlorophyll in fresh samples than in dried ones. The microalgal strains whose chlorophyll content was higher were F1, F2, and F3, especially in fresh biomass extracted with methanol and ethanol.

On the other hand, the quantification of carotenoids (Figure 1A) highlighted a clusterization that divided the fresh biomasses extracted with methanol and ethanol from all the other treatments. Accordingly, the fresh biomass of strains F3, F4, FB, and SEC_Li_ChL_1 extracted with methanol resulted in the greatest carotenoid content compared to the others, whereas, with the ethanol solvent, strains F2, F3, F4, C2, and R1 were those that produced greater amounts of carotenoids. On the other hand, the carotenoid yield was the lowest in strains F2 and CL_Sc under all the applied treatments/extractions, and the lowest in acetone and ethanol extraction in the dried biomass of all eleven strains.

### 3.3. Total Polyphenol and Flavonoid Content

Results of total polyphenol and flavonoid content in acetone, methanol, ethanol, and water extracts of both dry and fresh microalgal biomass are listed in Supplementary Tables S4 and S5, respectively. In general, from 1 to 100-fold higher total polyphenol content was detected in dry biomass extracts compared to the fresh ones, with the exclusion of F2 (methanol), F4 and CL_Sc (acetone), and C2 (ethanol). Moreover, as shown in Table S4, polyphenols were undetectable in fresh water biomass extracts, with the exclusion of F4, SEC_Li_ChL_1, CL_Sc, and Idr strains, where lower content than the dry biomass was detected. The highest polyphenol levels were determined in ethanolic and methanolic CL_Ch dry extracts (6.78 ± 1.51 and 5.88 ± 0.07 mg GAE g^−1^ dw, respectively) and methanolic and acetonic C2 dry extracts (6.41 ± 0.62 and 6.04 ± 0.21 mg GAE g^−1^ dw, respectively), followed by strains F2 (fresh biomass) and R1, F1, and FB (dry biomass) under methanol extraction. This evidence is also visible in the heatmap (Figure 1B), where it is also possible to observe the clusterization of the applied treatments. Indeed, the main clusterization divides dried from fresh samples, confirming the higher level of total polyphenols in dried samples than in fresh ones.

Regarding the flavonoid content, our data demonstrated from 1 to 30-fold greater flavonoid levels in dry biomasses than the fresh ones for methanolic, acetonic, and water extraction, with the exclusion of F2, F3, and CL_Ch acetone extracts (Table S5). Instead, for all algal strains, with the only exception of F1 and F2, opposite results were observed following ethanolic extraction, with higher flavonoid content in fresh biomass than in the dried ones. The highest flavonoid levels were detected in R1 and CL_Ch (128.9 ± 3.82 and 118.2 ± 4 mg QE g^−1^ dw, respectively) methanolic dry extracts, followed by strains C2 and F4 under the same treatment conditions. Moreover, similarly to total polyphenols, flavonoids were undetectable in fresh water biomass extracts, with the only exception of the C2 strain (Table S5). This evidence is also visible in the heatmap (Figure 1D), where it is possible to observe the separation of dry methanol and acetone extracts from the others, with the former containing greater amounts of total flavonoids, especially for strains R1, CL_Ch, C2, and F4.

### 3.4. In Vitro Antioxidant Activities

Results of FRAP and DPPH *in vitro* antioxidant activities of both dry and fresh microalgal biomass extracted in acetone, methanol, ethanol, and water are shown in Supplementary Figures S1 and S2, respectively.

**Figure 2:**
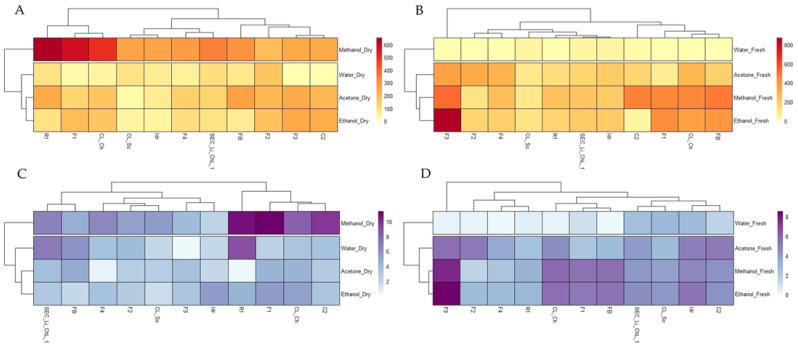
Heatmaps representing FRAP results of the dry (**A**) and fresh (**B**) biomass and DPPH results of the dry (**C**) and fresh (**D**) biomass of the microalgal strains under different types of solvent extraction. Each datum was obtained from the averaged values of three replicate measures.

Higher FRAP values were determined in methanol dry extracts, with the highest activity detected in R1 (683.04 ± 46.55 µM FeSO_4_), followed by the F1 (577.53 ± 36.43 µM FeSO_4_) and CL _Ch (509.31 ± 31.27 µM FeSO_4_) strains. This evidence is also visible in the heatmap (Figure 2A), where it is possible to observe the separation from the others of dry methanol extracts. Conversely, as evidenced in panel B of the heatmap (Figure 2B), FRAP activity was highest in the ethanol and methanol fresh biomass extracts and lowest in the water ones, with the highest values noted in the F3 ethanol (870.9 ± 0.00 µM FeSO_4_) and methanol (513.64 ± 0.09 µM FeSO_4_) extracts.

In agreement with the FRAP results, the highest ARA values, determined by the DPPH assay, were detected in methanol dry extracts and the lowest in the fresh water ones. Again, it is possible to observe a separation in the heatmap (Figure 2C,D) of the methanol dry and water fresh biomass extracts from others. In addition, as for FRAP results, the highest ARA values were detected in F1 (11.4 ± 0.45%), R1 (10.88 ± 0.35%), and C2 (9.76 ± 0.56%) dry methanol extracts and in F3 ethanol (8.55 ± 0.07%) and methanol (7.54 ± 0.22%) fresh biomass extracts.

### 3.5. Statistical Analysis Overview

**Figure 3:**
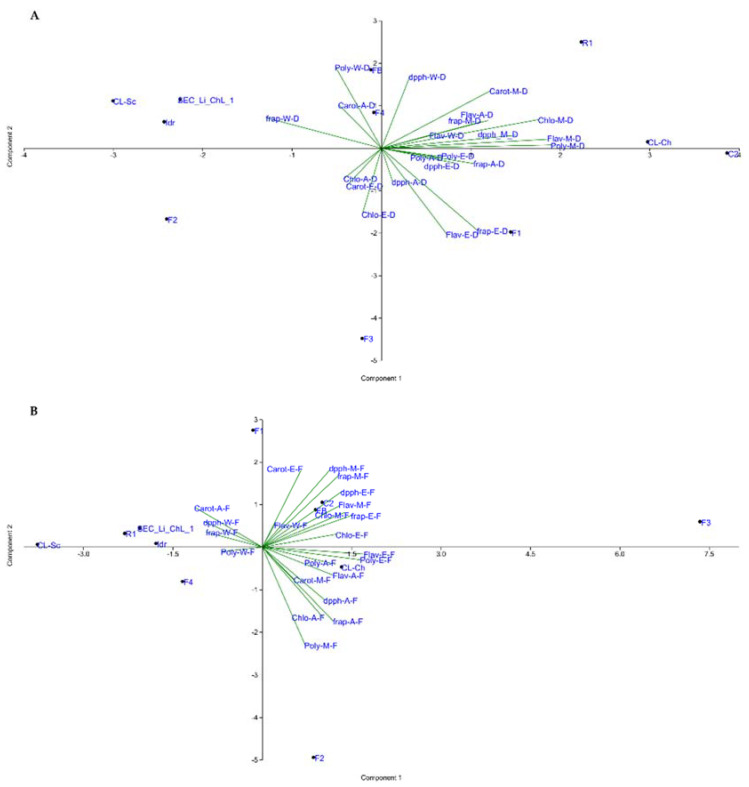
Principal component analysis (PCA) performed on the dataset for dry microalgal biomass (**A**) and fresh microalgal cultures (**B**), respectively.

Principal component analysis (PCA), performed separately on the dried (Figure 3A) and fresh (Figure 3B) biomass datasets, revealed two different outcomes. The main evidence in the PCA of the dried microalgal biomass (Figure 3A) is the position in the plot of strains C2 and CL_Ch, which are topologically located in opposition to strains F2, CL_Sc, Idr, and SEC_Li_ChL_1. Considering the fresh biomass, similar observations can be made on the PCA plot reported in Figure 3B, especially for strains F3 and CL_Sc, whose “isolated” positions in the plot stress their different characteristics compared to all the other strains. Moreover, strains F1 and F2 show opposite positions in the same plot (Figure 3B).

The correlation coefficients (R^2^) and relative *p*-values between carotenoids, polyphenols, flavonoids, FRAP, and DPPH data of both dried (Table 2) and fresh (Table 3) microalgal biomass were determined. As shown in Table 2, all analyzed parameters in dried microalgal biomass were positively correlated to each other.

Conversely, regarding fresh biomass, only the correlations between polyphenols and FRAP (*p* = 0.041), flavonoids and DPPH (*p* = 0.002), flavonoids and FRAP (*p* = 0.001), and FRAP and DPPH (*p* = 5.013 × 10^−11^) were found to be statistically significant.

## 4. Discussion

The increasing global population, as well as the growing attention of consumers to natural, sustainable, more nutritious, and healthier products, are driving both researchers and manufacturers and the food industry to search for alternative food sources [5,23,26]. In this context, microalgae, especially Spirulina (*Arthrospira* sp.) and *Chlorella* sp., are currently recognized as functional foods with important health-promoting effects [44].

In the food and pharmaceutical industry, microalgae can be used by adding them to common preparations such as cookies, pasta, bread, and cakes, or as a source of bioactive compounds, which can be purified and assumed as dietary supplements [45]. Although only a few species are currently consumed, many studies in the literature have aimed at evaluating other newly characterized species as possible candidates for functional foods and high-value products [7,46]. Our study aims at achieving the same objective, starting with the individuation of one or a few putative new microalgal candidates, isolated and characterized by natural local environments, to be potentially used in microalgal-based functional foods and pharmaceutical formulations.

In this work, eleven microalgal strains isolated from different freshwater environments were screened for different biochemical parameters, namely photosynthetic pigment content (total chlorophyll and carotenoids), total polyphenols and flavonoids, and for *in vitro* antioxidant activity. Both dried and fresh microalgal biomasses were investigated to highlight whether the applied pre-treatment might affect the content of molecules with nutraceutical potential.

According to the recent literature [16], different treatments of microalgal cultures are mandatory to optimize the extraction of functional molecules. In particular, before the extraction, the microalgal biomass needs to be harvested and preferably concentrated; sometimes, it is recommended to heat-treat the cells in order to induce their rupture. Therefore, in this work, we pre-treated the cells by harvesting and concentrating them by centrifugation. Moreover, a heat treatment was also applied to obtain dried biomass, as described in Section 2.3. According to literature data [47,48], the treatment of microalgal biomass with a temperature lower than 60 °C does not lead to photosynthetic pigment degradation.

Another factor affecting the differential production of molecules of nutraceutical interest in microalgal culture, including carotenoids, polyphenols, and flavonoids, is of course the choice of the culture medium, as well as the culture conditions [49]. Indeed, the composition of antioxidant compounds is strongly influenced by environmental stresses, nutrient availability, and environmental variables (light, temperature, pH, etc.) [22].

Interestingly, also the extraction method can affect the quality of molecules of nutraceutical interest, as well as their biological activity. Indeed, some conventional extraction procedures are time- and cost-consuming and are not able to preserve the residual biomass or the original characteristics of some extracted molecules. In the last several decades, many different extraction techniques have been developed and studied, with an increasing interest in those classified as “green” and “environmentally friendly” (for a review, please see [50]). These, namely comprehending switchable solvents, compressed fluid extractions, and microwave-assisted extraction, not only help to obtain different classes of molecules with high purity but also have a very low environmental impact.

In this work, we used the same growth medium and the same culture conditions (light and temperature) in order to minimize the effect of the environmental variables affecting the nutraceutical characterization of the different strains. These conditions were not necessarily stressful for our microalgal cultures, and, hence, they are probably not the optimal ones for the increased production of molecules of nutraceutical interest; nonetheless, these conditions are those that, up to now, have been applied for the maintenance of these collections.

In general, our data revealed the presence of variable content of chlorophyll and antioxidant compounds (total carotenoids, polyphenols, and flavonoids) and antioxidant activities, depending mainly on the solvent type and biomass treatment rather than on the geographical provenance of the microalgal strains or on their phylogenetic attribution. Consistently, the heatmaps confirmed this evidence (Figure 1 and Figure 2). Hence, in this work, the taxonomy seemed to not affect the different nutraceutical properties of the strains grown under the same conditions. Interestingly, in some cases, microalgae that belonged to the same species (e.g., F2 and F3, or FB, Idr, CL_Sc, and CL_Ch) showed contrasting behaviors and characteristics. This might suggest a kind of intraspecific variation, depending also on the differences occurring at the strain level, in agreement with Zhao et al. [11].

The quantification of chlorophyll among all the other biochemical parameters might be provide added value not only from a nutraceutical viewpoint. Accordingly, the recovery of chlorophyll to be used as a colorant or for its biotechnological potential might be coupled with the extraction of all the other biochemicals with nutraceutical potential, as discussed below. According to the literature, the application of different types of light for the growth of *Chlorella* sp. results in differences in chlorophyll production, varying from 0.74% of cell biomass under violet light to 1.29% of cell biomass under red light [51]. Moreover, the chlorophyll content in *C. vulgaris* can vary from 100 to 1500 mg L^−1^ in log and stationary phases [52].

In our experiment, the highest amount of chlorophyll was recovered by the fresh biomass of strain F3 extracted with methanol (approximately 3.84 µg mL^−1^ of fresh microalgal culture, corresponding to 3.84 mg L^−1^) and with ethanol (approximately 3.94 µg mL^−1^ of fresh microalgal culture, corresponding to 3.84 mg L^−1^). According to the yields of the fresh biomass shown in Table S1, in the former, the amount of chlorophyll corresponds to 0.022% of the dry weight, while, in the latter, it corresponds to 0.017%. Compared to the aforementioned literature [51,52], the chlorophyll content of our strain did not reach high values. This is probably due to the culturing conditions, which might not have stimulated our microalgae towards the massive production of photosynthetic pigments. Furthermore, the high yield of chlorophyll from fresh biomass with respect to the dry one was expected [47].

The accumulation of carotenoids within the microalgal cell allows the protection of the cell itself by storing energy and carbon. Among the most used microalgae for the production of carotenoids at the industrial level, we can list *Haematococcus pluvialis*, *Dunaliella salina*, *Chlorella* sp., *Scenedesmus* sp., and Spirulina [53].

Many authors have calculated the content of carotenoids produced by microalgae under different culture conditions [53,54]. According to our data, the highest carotenoid content was measured in the fresh biomass obtained from strain F3 (methanol and ethanol solvents) and strain F1 (ethanol solvent), corresponding to approximately 0.4 µg mL^−1^ of fresh microalgal culture. Considering the yield of each microalgal culture expressed as g dw fresh culture (Table S1), we can easily calculate that, for these two algae, the % of carotenoids with respect to the dry weight corresponds to less than 0.05% of the dry weight. According to the literature, the carotenoid content in microalgal cells might vary between 0.1 and 79% dry weight [53]. Hence, similarly to the chlorophyll content, we can suppose that the growing conditions to which we exposed our microalgal strains were not the most advantageous for the massive production of carotenoids. At the same time, we cannot a priori exclude the possibility that the application of stressful conditions (e.g., variations in the culture medium used, in the light, in the pH of the medium, etc.) might have affected the production of carotenoids in our strains.

Nowadays, microalgae are gaining much attention as “functional feedstocks” for foods, feeds, supplements, and nutraceutical formulations, being a source of high-value metabolites, including polyphenol compounds [5,23,26]. According to our results, the total polyphenol content of all microalgal extracts ranged from 0.015 to 6.782 mg GAE g^−1^ and from 0 to 4.856 mg GAE g^−1^ for dry and fresh biomasses, respectively (Table S4), with the highest values measured in CL_Ch (ethanolic and methanolic extracts) and C2 (methanolic and acetonic extracts) dry microalgal biomasses. Accordingly, other studies showed variable levels of total polyphenols depending on the algal strains and solvent extraction [12,27,55]. Our results are comparable to those obtained by Goiris et al. [12] on 32 microalgae strains (e.g., *Phaeodactylum tricornutum, Nannochloropsis* sp., *Isochrysis* sp., *Schizochytrium* sp., *Chlorella* sp., *Porphyridium cruentum*, *Botryococcus braunii, Tetraselmis* sp., and *Neochloris oleoabundans*) and those detected in *Chlorella vulgaris* and *Arthospira platensis* by Matos et al. [27], with values ranging from 0.54 to 4.57 mg GAE g^−1^ and 1.09 to 3.34 mg GAE g^−1^, respectively. Similarly, for 25 different algal species, Santhakumaran et al. [55] observed variable and sometimes higher levels of polyphenols, with values that varied from 0 to 54 mg GAE g^−1^, depending on the extracting solvents. Indeed, phenolic substances are strongly influenced by both solvent methods and environmental/growing conditions, which greatly affect, respectively, their extraction/solubility and production by microalgae [22].

Similarly to total polyphenols, microalgal strains contained variable levels of total flavonoids, with values ranging from 0 to 128.902 mg QE g^−1^ and from 0 to 86.433 mg QE g^−1^ for dry and fresh biomasses, respectively (Table S5), with the highest values detected in methanolic extracts obtained from R1 and CL_Ch dry biomasses. These results are comparable to those obtained by Santhakumaran et al. [55], where, under ethanol, methanol, and water extraction, the authors observed variable levels of flavonoids ranging from 0 to 136.22 mg QE g^−1^ dw, depending on the extracting solvents. Moreover, Tiong et al. [56] observed flavonoids values ranging from 14 to 34.7 mg QE g^−1^ in five Malaysian indigenous microalgae, namely *Auxenochlorella pyrenoidosa*, *Chlorella vulgaris*, *Messastrum gracile*, *Desmodesmus subspicatus,* and *Parachlorella kessleri*, which are covered in our range of values.

Interestingly, while photosynthetic pigments were extracted in higher amounts starting from fresh biomass in most cases, the total polyphenol and flavonoid (water, acetone, and methanol) content had the opposite behavior, showing the highest yields with dry biomass. Indeed, in general, from 1 to 100-fold and from 1 to 30-fold higher total polyphenol and flavonoid content, respectively, was found in dry biomass compared to the fresh ones. This was also highlighted by the heatmaps (Figure 2C,D), showing the clusterization of dried samples from the fresh ones, with the former containing greater amounts of total polyphenols, and the separation of dry methanol and acetone extracts from the others, with the latter containing lower flavonoid content. Moreover, regarding microalgal fresh biomass, our data pointed out the low extraction capacity of water, with total polyphenol and flavonoid values undetectable for the majority of strains.

The *in vitro* antioxidant activity of each microalgal strain, under different extraction and treatment conditions, was screened by FRAP and DPPH assays, expected to cover different mechanisms of antioxidant activity in the samples [11]. Indeed, while the former assesses the antioxidant power of microalgal strains to reduce, at low pH, ferric-tripyridyltriazine (Fe^3+^-TPTZ) to blue-colored ferrous-tripyridyltriazine complex (Fe^2+^-TPTZ), the latter is based on the reduction of the violet-colored DPPH radical to a pale-yellow-colored molecule by providing an electron or hydrogen atom [57].

According to our results, the microalgal strains contained antioxidant molecules that possess reducing power by donating both electrons and hydrogen atoms; moreover, for each assay, all microalgal strains exhibited antioxidant activity at different degrees, depending on both the treatment conditions and applied solvent. Specifically, regarding the FRAP assay, the antioxidant activity of microalgal extracts varied from 0 to 653.04 µM FeSO_4_ and from 0 to 870.9 µM FeSO_4_ for dry and fresh biomasses, respectively (Figure S1). These results are in line with those observed by Matos et al. [27] on *Chlorella vulgaris*, with FRAP activity of approximately 400 and 900 µM FeSO_4_ for aqueous and ethanolic extracts, respectively. Instead, as shown in Figure S2, the ARA values determined by the DPPH assay ranged from 0.16 to 11.4% radical inhibition for dry biomasses and from 0 to 8.55% for fresh ones. These results are lower than those obtained by Zhao et al. [11] in five freshwater microalgal strains, namely *Chlorella sorokiniana*, *Dictyosphaerium* sp., *Desmodesmus armatus*, *Scenedesmus ecornis*, and *Micractinium reisseri*, but higher than the DPPH activity detected in *Chlorella vulgaris* (ethanolic extract) and *Arthospira platensis* (aqueous and ethanolic extract) by Matos et al. [27], with the exclusion of an aqueous extract from *C. vulgaris*, showing very high % inhibition. According to our data, higher FRAP and DPPH values were found in the dry biomass obtained from methanol extracts of R1 and F1 and in fresh biomass from ethanol/methanol extracts of F3, while the lowest activities were detected in the water extracts.

All these data highlight different degrees of microalgae antioxidant activity that could depend on both biomass production and yield extraction or reflect potential limitations of conventional extraction procedures that, sometimes, could be not effective to preserve the biological activity of some extracted compounds [50]. Therefore, in the future, the use of new extraction strategies could represent a valuable alternative to some conventional techniques to reduce the time and improve the yields of extraction, and to preserve the biological activity of the extracted compounds.

The Pearson correlation results from dry microalgal biomass indicated that all the phytochemical compounds positively correlated with the antioxidant activities, suggesting that all of them contributed to the antioxidant properties of the tested microalgal strains. Conversely, FRAP and DPPH data in fresh microalgal biomass seemed to be linked to the flavonoid and polyphenol content instead of carotenoids. These results are in agreement with those reported by Goiris et al. [12] and by Hajimahmoodi et al. [58], showing positive correlations between carotenoids, phenolic content, and antioxidant activity or between phenolic content and FRAP, respectively. On the contrary, other studies showed insignificant correlations [22,59].

Finally, from the PCA analysis on the dried microalgal biomass (Figure 3A), the position of strains C2 and CL_Ch, in opposition to strains F2, CL_Sc, Idr, and SEC_Li_ChL_1, might be explained by the highest flavonoid, polyphenol, DPPH, and FRAP values detected in C2 and CL_Ch, in contrast to the lowest values measured in F2, CL_Sc, Idr, and SEC_Li_ChL_1 strains. According to the vectors in the PCA plot, these latter strains showed higher DPPH antiradical activity values with water extraction compared to CL_Ch and C2 (Figure 3A), in agreement with data shown in the relative heatmap (Figure 2A). Considering the fresh biomass (Figure 3B), similar considerations can be made regarding strains F3 and CL_Sc. Indeed, the “isolated” position of F3 in the plot reflects its ability to provide the highest amount of the different molecules in fresh biomass with respect to all the other strains. Interestingly, the opposite locations of strains F1 and F2 in the same plot might be attributed to the fact that the fresh biomass of strain F1 seemed to produce more carotenoids and displayed greater DPPH radical scavenging activity with respect to F2 (also confirmed in Figure 2), while the latter had higher polyphenol and chlorophyll content than F1.

## 5. Conclusions

Eleven different freshwater microalgal strains were screened for their nutraceutical properties. The strains belonged to the *Chlorella*-like clade (F4, R1, C2, FB, Idr, CL_Sc, CL_Ch, SEC_Li_ChL_1), to the family Scenedesmaceae (F1), and to the genus *Chlamydomonas* (F2 and F3). Although we are completely aware of the limitations of this study (e.g., the number of used solvents, the kind of extraction method based on chemical solvents, and the choice of the same growth conditions for all the strains), the results represent the first step of an explorative work aimed at selecting a few microalgal strains, for simultaneous massive growth and bioactive compound production, that will be cultivated in the future under different conditions and tested for their nutraceutical properties with different extraction methods. Moreover, this study offers a new perspective for the selection of one or a few newly characterized microalgal strains to apply in high-density cultivation as sources of bioactive compounds for food/feed and pharmaceuticals. Results highlighted different characteristics of each strain that were not determined by the taxonomic affiliation or by the isolation source, but mainly by the treatment (e.g., fresh vs. dry biomass) and the extraction conditions (different kinds of solvents). According to the results, the putative best candidates for massive cultivation under laboratory conditions for the simultaneous extraction of different molecules with nutraceutical potential are strains F1 (Scenedesmaceae), F3 (*Chlamydomonas debariana*), R1 (*Chlorella sorokiniana*), and C2 (*Chlorella-*like). Further studies focused on the cultivation of the selected microalgae under stressful conditions will be explored to understand the impact of the environmental variables and maximize the bioactive compounds’ content. Moreover, the potential application of the investigated fraction in new food and pharmaceutical formulations will be explored for microalgae-based product development. Finally, the use of new and green extraction strategies could represent, in future studies, a valuable alternative to conventional techniques to reduce the time and improve the yields of extraction.

## Figures and Tables

**Table 1:** Taxonomic affiliation of the six microalgal strains newly isolated from regional freshwater environments.

Strain	Isolation Source	Sequenced Gene	Accession Number	Length (bp)	Best Blast Hit (Identity %)	Taxonomic Affiliation
F1	“Le Morette”, Fucecchio Marshland	18S rDNA	OM311002	2919	*Desmodesmus brasiliensis* AB917106 (94.53)	Scenedesmaceae
		Final portion of 18S, complete ITS1-5.8S-ITS2, initial portion of 28S rDNA	OM310999	1153	*Desmodesmus subspicatus* MK975481 (96.63)	
F2	“Le Morette”, Fucecchio Marshland	18S rDNA	OM311003	1703	*Chlamydomonas debaryana* MF678003 (99.82)	*Chlamydomonas debaryana*
F3	“Le Morette”, Fucecchio Marshland	18S rDNA	OM311004	1716	*Chlamydomonas debaryana* MF678003 (99.94)	*Chlamydomonas debaryana*
F4	“Le Morette”, Fucecchio Marshland	18S rDNA	OM311005	1758	*Chlorella sorokiniana* MF101221 (100)	*Chlorella sorokiniana*
		Final portion of 18S, complete ITS1-5.8S-ITS2, initial portion of 28S rDNA	OM311000	738	*Chlorella sorokiniana* KM514859 (99.59)	
R1	Private terrace in Pisa, water sample	18S rDNA	OM311006	1758	*Chlorella sorokiniana* MF101221 (99.89)	*Chlorella sorokiniana*
C2	Heating system of a private house	18S rDNA	OM311001	1704	*Chlorella* sp. KP262476 (99.30)	*Chlorella-*like microalgal strain
		Final portion of 18S, complete ITS1-5.8S-ITS2, initial portion of 28S rDNA	OM310998	1279	*Chlorella vulgaris* KT778121 (99.70)	

**Table 2:** Pearson correlation analysis performed on carotenoids, polyphenols, flavonoids, FRAP, and DPPH data of dry microalgal biomass.

*p*-Value	Carotenoids	Polyphenols	Flavonoids	DPPH	FRAP
R^2^					
Carotenoids		0.021	2.51 × 10^−4^	4.12 × 10^−5^	6.46 × 10^−5^
Polyphenols	0.400		2.32 × 10^−4^	2.7 × 10^−4^	5.45 × 10^−4^
Flavonoids	0.596	0.539		0.004	4.31 × 10^−10^
DPPH	0.651	0.523	0.430		4.27 × 10^−6^
FRAP	0.638	0.506	0.798	0.638	

Correlation coefficients (R^2^) and *p*-values are listed on the left and the right of the table, respectively.

**Table 3:** Pearson correlation analysis performed on carotenoids, polyphenols, flavonoids, FRAP, and DPPH data of fresh microalgal biomass.

*p*-Value	Carotenoids	Polyphenols	Flavonoids	DPPH	FRAP
R^2^					
Carotenoids		0.738	0.939	0.624	0.41
Polyphenols	−0.061		0.065	0.393	0.041
Flavonoids	−0.014	0.3259		0.002	0.001
DPPH	0.089	0.147	0.505		5.013 × 10^−11^
FRAP	0.1489	0.337	0.549	0.827	

Correlation coefficients (R^2^) and *p*-values are listed on the left and the right of the table, respectively.

## Data Availability

All data generated or analyzed in this study are included in the published article. The 18S rDNA and ITS sequences have been deposited in GenBank and accession numbers are shown in the Results section, in Table 1 of the present manuscript.

## Supplementary Materials

The following are available online at https://www.mdpi.com/article/10.3390/foods11050654/s1, Table S1. The dry biomass weight (yield) of 15 mL of each fresh microalgal culture. Table S2. Total chlorophyll content of dry and fresh microalgal strains, under different types of solvent extraction, expressed as µg ml^−1^ of fresh culture volume. Each datum is the mean ± SD from three replicates. Table S3. Carotenoid content of dry and fresh microalgal strains, under different types of solvent extraction, expressed as µg ml^−1^ of fresh culture volume. Each datum is the mean ± SD from three replicates. Table S4. Total polyphenol content of dry and fresh microalgal strains, under different types of solvent extraction, expressed as mg GAE g^−1^ dw. Fresh microalgal results were calculated based on the yield listed in Table S1. Each datum is the mean ± SD from three replicates. Table S5. Total flavonoid content of dry and fresh microalgal strains, under different types of solvent extraction, expressed as mg QE g^−1^ dw. Fresh microalgal results were calculated based on the yield listed in Table S1. Each datum is the mean ± SD from three replicates. Figure S1. FRAP activity, expressed as μM FeSO_4_, of 85 μL extract of dry and fresh microalgal biomass under different types of solvent extraction. Each datum is the mean ± SD from three replicates. Figure S2. The antiradical activity (ARA), expressed as a percentage of DPPH inhibition, of 50 μL extract of dry and fresh microalgal biomass under different types of solvent extraction. Each datum is the mean ± SD from three replicates.

## References

[1] De Souza M.P., Hoeltz M., Gressler P.D., Benitez L.B., Schneider R.C.S. (2019). Potential of Microalgal Bioproducts: General Perspectives and Main Challenges. Waste Biomass Valoriz..

[2] Mehariya S., Goswami R.K., Karthikeysan O.P., Verma P. (2021). Microalgae for high-value products: A way towards green nutraceutical and pharmaceutical compounds. Chemosphere.

[3] Richmond A. (2008). Handbook of Microalgal Culture: Biotechnology and Applied Phycology.

[4] Colla E., Menegotto A.L.L., Kalschne D.L., da Silva-Buzanello R.A., Canan C., Drunkler D.A., Konur O. (2020). Chapter 32-Microalgae: A new and promising source of food. Handbook of Algal Science, Technology and Medicine.

[5] Galasso C., Gentile A., Orefice I., Ianora A., Bruno A., Noonan D.M., Sansone C., Albini A., Brunet C. (2019). Microalgal Derivatives as Potential Nutraceutical and Food Supplements for Human Health: A Focus on Cancer Prevention and Interception. Nutrients.

[6] Udayan A., Arumugam M., Pandey A., Rastogi R.P., Madamwar D., Pandey A. (2017). Chapter 4-Nutraceuticals From Algae and Cyanobacteria. Algal Green Chemistry.

[7] Molino A., Iovine A., Casella P., Mehariya S., Chianese S., Cerbone A., Rimauro J., Musmarra D. (2018). Microalgae Characterization for Consolidated and New Application in Human Food, Animal Feed and Nutraceuticals. Int. J. Environ. Res. Public Health.

[8] García J.L., de Vicente M., Galán B. (2017). Microalgae, old sustainable food and fashion nutraceuticals. Microb. Biotechnol..

[9] Sousa I., Gouveia L., Batista A., Raymundo A., Bandarra N. (2008). Chapter 2. Microalgae in novel food products. Algae Nutr. Pollut. Control. Energy Sources.

[10] Gabriele M., Pucci L. (2017). Diet Bioactive Compounds: Implications for Oxidative Stress and Inflammation in the Vascular System. Endocr. Metab. Immune Disord. Drug Targets.

[11] Zhao Z., Rasool M.A., Chen C., Ma S., Wang L., Huang G. (2020). Identification and screening of multiple tropical microalgal strains for antioxidant activity in vitro. Food Biosci..

[12] Goiris K., Muylaert K., Fraeye I., Foubert I., De Brabanter J., De Cooman L. (2012). Antioxidant potential of microalgae in relation to their phenolic and carotenoid content. J. Appl. Phycol..

[13] Sharifi-Rad M., Anil Kumar N.V., Zucca P., Varoni E.M., Dini L., Panzarini E., Rajkovic J., Tsouh Fokou P.V., Azzini E., Peluso I. (2020). Lifestyle, Oxidative Stress, and Antioxidants: Back and Forth in the Pathophysiology of Chronic Diseases. Front. Physiol..

[14] Jerez-Martel I., García-Poza S., Rodríguez-Martel G., Rico M., Afonso-Olivares C., Gómez-Pinchetti J.L. (2017). Phenolic Profile and Antioxidant Activity of Crude Extracts from Microalgae and Cyanobacteria Strains. J. Food Qual..

[15] Nicoletti M. (2016). Microalgae Nutraceuticals. Foods.

[16] Yatipanthalawa B., Martin G., Lafarga T., Acién G. (2021). Chapter 3-Conventional and novel approaches to extract food ingredients and nutraceuticals from microalgae. Cultured Microalgae for the Food Industry.

[17] Castro-Muñoz R., García-Depraect O. (2021). Membrane-Based Harvesting Processes for Microalgae and Their Valuable-Related Molecules: A Review. Membranes.

[18] Da Silva Ferreira V., Sant’Anna C. (2017). Impact of culture conditions on the chlorophyll content of microalgae for biotechnological applications. World J. Microbiol. Biotechnol..

[19] Timberlake C.F., Henry B.S. (1986). Plant pigments as natural food colours. Endeavour.

[20] Vesenick D., Paula N., Niwa A., Mantovani M. (2012). Evaluation of the Effects of Chlorophyllin on Apoptosis Induction, Inhibition of Cellular Proliferation and mRNA Expression of CASP8, CASP9, APC and $-catenin. Curr. Res. J. Biol. Sci..

[21] Cezare-Gomes E.A., Mejia-da-Silva L.d.C., Pérez-Mora L.S., Matsudo M.C., Ferreira-Camargo L.S., Singh A.K., de Carvalho J.C.M. (2019). Potential of Microalgae Carotenoids for Industrial Application. Appl. Biochem. Biotechnol..

[22] Maadane A., Merghoub N., Tarik A., El Arroussi H., Benhima R., Amzazi S., Bakri Y., Wahby I. (2015). Antioxidant activity of some Moroccan marine microalgae: Pufa profiles, carotenoids and phenolic content. J. Biotechnol..

[23] Conde T.A., Neves B.F., Couto D., Melo T., Neves B., Costa M., Silva J., Domingues P., Domingues M.R. (2021). Microalgae as Sustainable Bio-Factories of Healthy Lipids: Evaluating Fatty Acid Content and Antioxidant Activity. Mar. Drugs.

[24] Kesavan P., Banerjee A., Banerjee A., Murugesan R., Marotta F., Pathak S. (2018). Chapter 17. An Overview of Dietary Polyphenols and Their Therapeutic Effects. Polyphen. Mech. Action Hum. Health Dis..

[25] Freile-Pelegrin Y., Robledo D. (2014). Chapter 6. Bioactive phenolic compounds from algae. Bioact. Compd. Mar. Foods Plant. Anim. Sources.

[26] Sansone C., Brunet C. (2019). Promises and Challenges of Microalgal Antioxidant Production. Antioxidants.

[27] Matos J., Cardoso C.L., Falé P., Afonso C.M., Bandarra N.M. (2020). Investigation of nutraceutical potential of the microalgae Chlorella vulgaris and Arthrospira platensis. Int. J. Food Sci. Technol..

[28] Islam M.N., Alsenani F., Schenk P. (2017). Chapter 1. Microalgae as a Sustainable Source of Nutraceuticals. Microb. Funct. Foods Nutraceuticals.

[29] Camacho F., Macedo A., Malcata F. (2019). Potential Industrial Applications and Commercialization of Microalgae in the Functional Food and Feed Industries: A Short Review. Mar. Drugs.

[30] Patel A.K., Singhania R.R., Awasthi M.K., Varjani S., Bhatia S.K., Tsai M.-L., Hsieh S.-L., Chen C.-W., Dong C.-D. (2021). Emerging prospects of macro- and microalgae as prebiotic. Microb. Cell Factories.

[31] Lauritano C., Andersen J.H., Hansen E., Albrigtsen M., Escalera L., Esposito F., Helland K., Hanssen K.Ø., Romano G., Ianora A. (2016). Bioactivity Screening of Microalgae for Antioxidant, Anti-Inflammatory, Anticancer, Anti-Diabetes, and Antibacterial Activities. Front. Mar. Sci..

[32] Chiellini C., Guglielminetti L., Sarrocco S., Ciurli A. (2020). Isolation of Four Microalgal Strains From the Lake Massaciuccoli: Screening of Common Pollutants Tolerance Pattern and Perspectives for Their Use in Biotechnological Applications. Front. Plant Sci..

[33] Ciurli A., Modeo L., Pardossi A., Chiellini C. (2021). Multidisciplinary integrated characterization of a native Chlorella-like microalgal strain isolated from a municipal landfill leachate. Algal Res..

[34] Gorman D.S., Levine R.P. (1965). Cytochrome f and plastocyanin: Their sequence in the photosynthetic electron transport chain of Chlamydomonas reinhardi. Proc. Natl. Acad. Sci. USA.

[35] Saba F., Papizadeh M., Khansha J., Sedghi M., Rasooli M., Amoozegar M., Soudi M., Shahzadeh Fazeli S.A. (2016). A Rapid and Reproducible Genomic DNA Extraction Protocol for Sequence-Based Identification of Archaea, Bacteria, Cyanobacteria, Diatoms, Fungi, and Green Algae. J. Med. Bacteriol..

[36] Altschul S.F., Madden T.L., Schäffer A.A., Zhang J., Zhang Z., Miller W., Lipman D.J. (1997). Gapped BLAST and PSI-BLAST: A new generation of protein database search programs. Nucleic Acids Res..

[37] Lichtenthaler H.K. (1987). [34] Chlorophylls and carotenoids: Pigments of photosynthetic biomembranes. Methods in Enzymology.

[38] Gabriele M., Parri E., Felicioli A., Sagona S., Pozzo L., Biondi C., Domenici V., Pucci L. (2015). Phytochemical Composition and Antioxidant Activity of Tuscan Bee Pollen of Different Botanic Origins. Ital. J. Food Sci..

[39] Colosimo R., Gabriele M., Cifelli M., Longo V., Domenici V., Pucci L. (2020). The effect of sourdough fermentation on Triticum dicoccum from Garfagnana: ^1^H NMR characterization and analysis of the antioxidant activity. Food Chem..

[40] Boudjou S., Oomah B.D., Zaidi F., Hosseinian F. (2013). Phenolics content and antioxidant and anti-inflammatory activities of legume fractions. Food Chem..

[41] Team R Core (2013). R: A Language and Environment for Statistical Computing.

[42] Kolde R. (2019). Pheatmap: Pretty Heatmaps. https://CRAN.R-project.org/package=pheatmap.

[43] Hammer O., Harper D., Ryan P. (2001). PAST: Paleontological Statistics Software Package for Education and Data Analysis. Palaeontol. Electron..

[44] Koyande A.K., Chew K.W., Rambabu K., Tao Y., Chu D.-T., Show P.-L. (2019). Microalgae: A potential alternative to health supplementation for humans. Food Sci. Hum. Wellness.

[45] Alam M.A., Xu J.-L., Wang Z. (2020). Microalgae Biotechnology for Food, Health and High Value Products.

[46] Bao B., Thomas-Hall S.R., Schenk P.M. (2022). Fast-Tracking Isolation, Identification and Characterization of New Microalgae for Nutraceutical and Feed Applications. Phycology.

[47] Sarkar S., Manna M.S., Bhowmick T.K., Gayen K. (2020). Extraction of chlorophylls and carotenoids from dry and wet biomass of isolated Chlorella Thermophila: Optimization of process parameters and modelling by artificial neural network. Process. Biochem..

[48] Pasquet V., Chérouvrier J.-R., Farhat F., Thiéry V., Piot J.-M., Bérard J.-B., Kaas R., Serive B., Patrice T., Cadoret J.-P. (2011). Study on the microalgal pigments extraction process: Performance of microwave assisted extraction. Process. Biochem..

[49] Chia M., Lombardi A., Melão M. (2013). Growth and biochemical composition of Chlorella vulgaris in different growth media. An. Da Acad. Bras. De Cienc..

[50] Imbimbo P., D’Elia L., Liberti D., Olivieri G., Monti D.M. (2020). Towards green extraction methods from microalgae learning from the classics. Appl. Microbiol. Biotechnol..

[51] Mohsenpour S.F., Richards B., Willoughby N. (2012). Spectral conversion of light for enhanced microalgae growth rates and photosynthetic pigment production. Bioresour. Technol..

[52] Rinawati M., Sari L., Pursetyo K. (2020). Chlorophyll and carotenoids analysis spectrophotometer using method on microalgae. IOP Conf. Ser. Earth Environ. Sci..

[53] Ambati R.R., Gogisetty D., Aswathanarayana R.G., Ravi S., Bikkina P.N., Bo L., Yuepeng S. (2019). Industrial potential of carotenoid pigments from microalgae: Current trends and future prospects. Crit. Rev. Food Sci. Nutr..

[54] Guedes A.C., Amaro H.M., Malcata F.X. (2011). Microalgae as sources of carotenoids. Mar. Drugs.

[55] Santhakumaran P., Ayyappan S.M., Ray J.G. (2020). Nutraceutical applications of twenty-five species of rapid-growing green-microalgae as indicated by their antibacterial, antioxidant and mineral content. Algal Res..

[56] Tiong I.K.R., Nagappan T., Abdul Wahid M.E., Tengku Muhammad T.S., Tatsuki T., Satyantini W.H., Mahasri G., Sorgeloos P., Sung Y.Y. (2020). Antioxidant capacity of five microalgae species and their effect on heat shock protein 70 expression in the brine shrimp Artemia. Aquac. Rep..

[57] Mfotie Njoya E. (2021). Medicinal Plants, Antioxidant Potential, and Cancer.

[58] Hajimahmoodi M., Faramarzi M., Mohammadi N., Soltani N., Oveisi M.R., Nafissi-Varcheh N. (2010). Evaluation of antioxidant properties and total phenolic contents of some strains of microalgae. J. Appl. Phycol..

[59] Goh S.-H., Yusoff F.M., Loh S.P. (2010). A Comparison of the Antioxidant Properties and Total Phenolic Content in a Diatom, *Chaetoceros* sp. and a Green Microalga, *Nannochloropsis* sp. J. Agric. Sci..

