# Supplementary material for: Evaluation of Nutraceutical Properties of Eleven Microalgal Strains Isolated from Different Freshwater Aquatic Environments: Perspectives for Their Application as Nutraceuticals
Source: Foods. 2022 Feb 23;11(5):654. doi: 10.3390/foods11050654 (PMC8909373; doi:10.3390/foods11050654)
Supplement: Supplementary file 1 [file foods-11-00654-s001.zip › foods-1586466-Supplementary materials.pdf]

Supplementary materials

Table S1. The dry biomass weight (yield) of 15 ml of each fresh microalgal culture.

**Yield (g)**	**F1**	**F2**	**F3**	**F4**	**R1**	**SEC_Li_ChL_1**	**CL_Sc**	**CL_Ch**	**Idr**	**FB**	**C2**
Acetone	0.018	0.015	0.007	0.018	0.022	0.046	0.012	0.0121	0.017	0.0027	0.016
Methanol	0.021	0.001	0.017	0.009	0.016	0.009	0.008	0.0117	0.008	0.0139	0.006
Ethanol	0.027	0.006	0.023	0.009	0.012	0.013	0.010	0.0115	0.010	0.0145	0.007
Water	0.013	0.002	0.004	0.020	0.012	0.012	0.034	0.0132	0.005	0.022	0.008

Table S2. Total chlorophyll content of dry and fresh microalgal strains, under different types of solvent extraction, expressed as μg ml^{-1} of fresh culture volume. Each datum is the mean ± SD from three replicates.

**Total Chlorophyll**		F1	F2	F3	F4	R1	C2	SEC_Li_ChL_1	CL_Sc	CL_Ch	Idr	FB
Acetone	*Dry*	0.24±1.7 × 10^{-4}	0.12±1.0 × 10^{-3}	0.35±3.2 × 10^{-5}	0.1±9.8 × 10^{-5}	0.26±3.0 × 10^{-4}	0.09±5.5 × 10^{-5}	0.24±4.5 × 10^{-5}	0.10±4.0 × 10^{-5}	0.09±5.0 × 10^{-5}	0.31±2.1 × 10^{-5}	0.21±1.2 × 10^{-4}
	Fresh	0.83±1.4 × 10^{-3}	2.68±2.2 × 10^{-4}	2.66±4.1 × 10^{-4}	1.78±6.8 × 10^{-5}	1.64±9.2 × 10^{-4}	1.23±9.8 × 10^{-5}	1.07±3.6 × 10^{-4}	0.61±1.8 × 10^{-4}	0.82±1.6 × 10^{-4}	1.37±1.1 × 10^{-4}	1.03±2.7 × 10^{-4}
Methanol	*Dry*	1.03±3.5 × 10^{-4}	0.36±1.4 × 10^{-4}	0.68±1.4 × 10^{-3}	1.01±1.2 × 10^{-5}	1.11±7.9 × 10^{-5}	1.11±1.0 × 10^{-4}	0.74±1.6 × 10^{-4}	0.48±3.9 × 10^{-5}	1.16±2.8 × 10^{-4}	0.42±1.1 × 10^{-5}	1.12±4.8 × 10^{-4}
	Fresh	2.75±7.9 × 10^{-4}	1.76±2.6 × 10^{-4}	3.84±2.1 × 10^{-3}	1.82±1.9 × 10^{-4}	1.42±7.0 × 10^{-4}	2.13±3.1 × 10^{-5}	1.87±3.1 × 10^{-5}	0.85±1.0 × 10^{-4}	1.13±2.0 × 10^{-4}	1.88±1.3 × 10^{-3}	1.60±1.5 × 10^{-4}
Ethanol	*Dry*	0.40±3.8 × 10^{-4}	0.31±2.6 × 10^{-5}	0.44±2.6 × 10^{-5}	0.22±1.6 × 10^{-4}	0.3±4.1 × 10^{-5}	0.13±2.8 × 10^{-5}	0.28±2.8 × 10^{-5}	0.14±6.1 × 10^{-6}	0.09±1.5 × 10^{-4}	0.16±3.5 × 10^{-5}	0.16±2.4 × 10^{-4}
	Fresh	3.01±3.0 × 10^{-4}	2.33±1.0 × 10^{-4}	3.94±1.5 × 10^{-3}	1.51±2.0 × 10^{-4}	1.99±2.3 × 10^{-4}	1.54±7.8 × 10^{-5}	1.71±1.7 × 10^{-4}	0.88±2.0 × 10^{-4}	1.12±3.3 × 10^{-4}	1.86±1.9 × 10^{-4}	1.29±2.0 × 10^{-5}

Table S3. Carotenoids content of dry and fresh microalgal strains, under different types of solvent extraction, expressed as μg ml^{-1} of fresh culture volume. Each datum is the mean ± SD from three replicates.

**Carotenoids**		F1	F2	F3	F4	R1	C2	SEC_Li_ChL_1	CL_Sc	CL_Ch	Idr	FB
Acetone	*Dry*	0.04±7.1 × 10^{-5}	0.02±2.7 × 10^{-4}	0.04±1.8 × 10^{-5}	0.02±2.3 × 10^{-5}	0.06±1.6 × 10^{-4}	0.03±7.55 × 10^{-5}	0.07±1.5 × 10^{-5}	0.04±1.5 × 10^{-5}	0.03±7.1 × 10^{-6}	0.08±2.7 × 10^{-4}	0.06±9.2 × 10^{-5}
	Fresh	0.20±6.9 × 10^{-4}	0.07±4.4 × 10^{-4}	0.08±3.3 × 10^{-4}	0.29±4.3 × 10^{-5}	0.32±5.8 × 10^{-4}	0.12±2.31 × 10^{-5}	0.28±6.7 × 10^{-5}	0.2±4.9 × 10^{-5}	0.23±7.6 × 10^{-5}	0.31±2.2 × 10^{-4}	0.29±3.0 × 10^{-5}
Methanol	*Dry*	>0.2	0.01±3.0 × 10^{-5}	0.002±8.1 × 10^{-4}	>0.2	>0.2	>0.2	0.04±3.0 × 10^{-4}	0.13±1.0 × 10^{-4}	>0.2	0.16±1.5 × 10^{-4}	>0.2
	Fresh	0.05±7.7 × 10^{-4}	0.24±4.6 × 10^{-4}	>0.4	0.36±5.4 × 10^{-4}	0.20±5.0 × 10^{-4}	0.16±5.63 × 10^{-4}	0.35±5.8 × 10^{-4}	0.24±6.0 × 10^{-5}	0.31±2.1 × 10^{-3}	0.29±4.8 × 10^{-4}	0.35±7.7 × 10^{-4}
Ethanol	*Dry*	0.12±2.1 × 10^{-4}	0.05±2.3 × 10^{-5}	0.07±3.8 × 10^{-5}	0.06±9.5 × 10^{-5}	0.5±8.0 × 10^{-5}	0.03±1.09 × 10^{-4}	0.08±2.2 × 10^{-5}	0.05±4.2 × 10^{-6}	0.03±1.9 × 10^{-5}	0.05±6.1 × 10^{-5}	0.06±1.0 × 10^{-5}
	Fresh	>0.4	0.19±4.3 × 10^{-4}	>0.4	0.38±2.6 × 10^{-4}	0.34±1.2 × 10^{-4}	0.38±3.73 × 10^{-4}	0.31±3.3 × 10^{-4}	0.20±5.6 × 10^{-5}	0.25±6.9 × 10^{-5}	0.27±2.7 × 10^{-4}	0.28±8.0 × 10^{-5}

Table S4. Total polyphenols content of dry and fresh microalgal strains, under different types of solvent extraction, expressed as mg GAE g^{-1} dw. Fresh microalgal results were calculated based on the yield listed in Table S1. Each datum is the mean ± SD from three replicates.

**Total polyphenols**		**F1**	**F2**	**F3**	**F4**	**R1**	**SEC_Li_ChL_1**	**CL_Sc**	**CL_Ch**	**Idr**	**FB**	**C2**
Acetone	*Dry*	0.20±0.06	2.61±0.01	2.81±0.42	0.08±1 × 10^{-3}	1.68±0.11	1.20±0.20	0.14±0.05	1.67±0.12	3.22±0.51	3.45±0.38	6.04±0.21
	Fresh	0.18±0.01	1.41±0.02	2.50±0.46	0.63±0.15	0.13±0.05	0.37±0.01	0.67±0.05	1.18±0.08	0.82±0.07	3.01±0.43	0.61±0.01
Methanol	*Dry*	4.08±0.11	2.01±4 × 10^{-3}	3.30±0.01	2.51±0.03	4.13±0.08	2.80±0.23	2.17±0.15	5.88±0.07	2.21±0.25	4.05±0.10	6.41±0.62
	Fresh	0.66±4 × 10^{-3}	4.85±1.12	1.83±0.25	0.63±0.05	0.43±0.12	1.06±0.01	0.59±0.07	1.68±0.11	1.18±0.08	1.37±0.11	1.66±0.07
Ethanol	*Dry*	1.29±0.24	1.92±0.05	2.32±0.20	0.99±0.29	0.74±0.09	0.24±2 × 10^{-3}	1.34±0.27	6.78±1.51	0.55±0.07	3.84±0.99	0.76±0.39
	Fresh	0.81±0.03	1.23±0.21	2.08±0.06	0.26±0.10	0.20±1 × 10^{-3}	0.20±0.06	0.56±0.02	1.39±0.04	0.31±0.08	0.81±0.07	1.27±0.16
Water	*Dry*	0.42±0.01	1.80±0.12	0.02±4 × 10^{-3}	2.27±0.01	2.72±0.16	3.65±0.05	2.27±0.07	0.96±0.05	1.11±0.09	3.80±0.08	1.52±0.05
	Fresh	n.d.	n.d.	n.d.	0.08±0.01	n.d.	0.03±0.02	0.02±2 × 10^{-3}	n.d.	0.15±0.01	n.d.	n.d.

n.d. not detected

Table S5. Total flavonoids content of dry and fresh microalgal strains, under different types of solvent extraction, expressed as mg QE g-1 dw. Fresh microalgal results were calculated based on the yield listed in Table S1. Each datum is the mean ± SD from three replicates.

**Total flavonoids**		**F1**	**F2**	**F3**	**F4**	**R1**	**SEC_Li_ChL_1**	**CL_Sc**	**CL_Ch**	**Idr**	**FB**	**C2**
Acetone	*Dry*	19.98±0.28	15.11±5.93	38.37±8.12	45.04±0.04	88.09±13.91	20.77±3.31	6.88±1.98	28.61±1.37	60.61±4.57	34.94±0.73	89.06±5.01
	Fresh	2.46±0.01	27.38±1.13	58.32±2.72	12.73±0.05	2.89±0.09	3.55±0.06	5.70±1.36	86.43±7.98	8.86±0.55	9.81±0.50	13.78±0.64
Methanol	*Dry*	83.71±3.69	38.81±2.08	74.60±2.32	95.33±0.98	128.90±3.82	49.07±1.25	36.39±2.51	118.20±3.99	27.27±0.10	55.36±0.49	106.51±2.64
	Fresh	16.95±0.31	11.88±1.83	53.02±1.14	21.35±2.41	13.18±1.17	23.23±1.53	11.45±0.63	27.70±0.65	24.33±1.42	38.02±2.72	57.62±1.25
Ethanol	*Dry*	24.72±0.45	34.97±0.53	49.19±2.85	14.38±0.64	16.83±1.56	10.02±1.37	5.20±1.36	19.32±0.35	4.30±0.49	9.80±0.04	35.06±3.15
	Fresh	16.59±0.34	29.22±2.18	51.24±1.52	16.57±1.52	16.18±1.57	12.25±0.40	7.01±0.20	23.44±0.99	16.96±0.71	29.49±0.30	37.92±0.18
Water	*Dry*	n.d.	3.22±0.68	n.d.	0.45±0.19	1.58±0.63	5.93±0.20	9.92±0.25	0.29±0.06	1.78±0.50	2.42±0.40	25.76±2.61
	Fresh	n.d.	n.d.	n.d.	n.d.	n.d.	n.d.	n.d.	n.d.	n.d.	n.d.	15.06±0.92

n.d. not detected

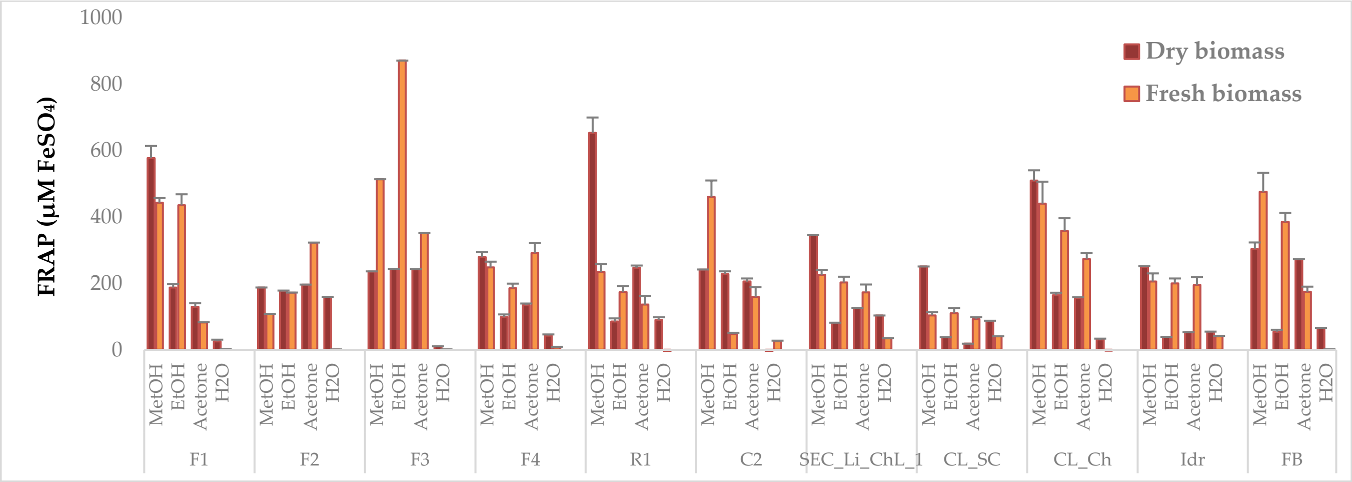

Figure S1. FRAP activity, expressed as μM $FeSO_4$, of 85 μL extract of dry and fresh microalgal biomass under different types of solvent extraction. Each datum is the mean ± SD from three replicates.

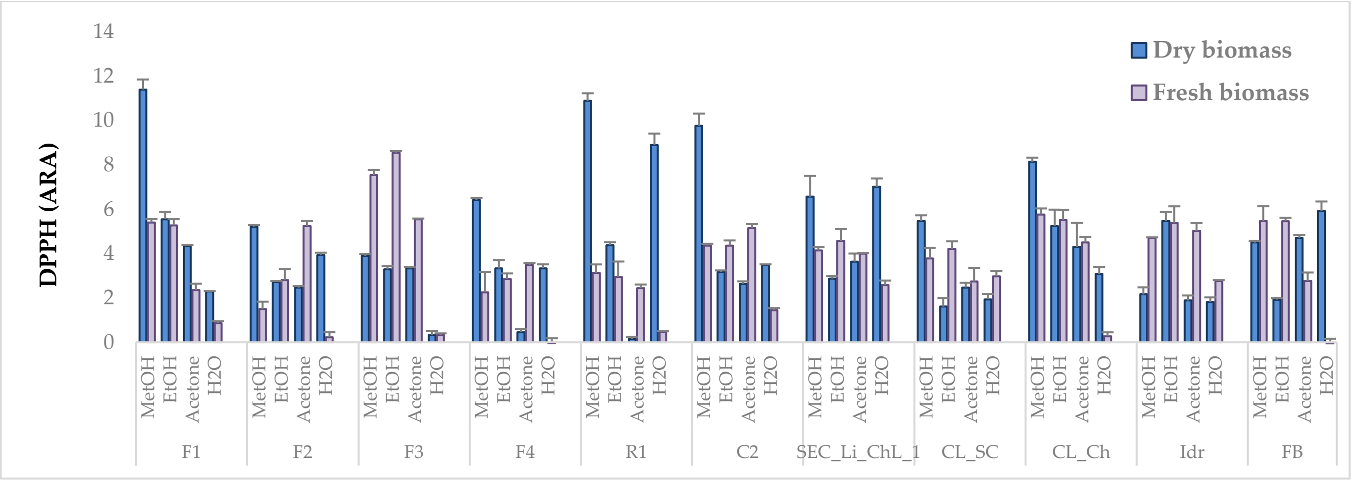

Figure S2. The antiradical activity (ARA), expressed as a percentage of DPPH inhibition, of 50 μL extract of dry and fresh microalgal biomass under different types of solvent extraction. Each datum is the mean ± SD from three replicates.
